# Candida Bloodstream Infections and Associated Risk Factors in Pediatric Cardiac Intensive Care

**DOI:** 10.3390/diagnostics15081001

**Published:** 2025-04-14

**Authors:** Onur Ozalp, Erkut Ozturk

**Affiliations:** 1 Department of Infectious Diseases and Clinical Microbiology, Basaksehir Cam and Sakura Hospital, University of Health Sciences, Istanbul 34480, Turkey; 2 Department of Pediatric Cardiology, Basaksehir Cam and Sakura Hospital, University of Health Sciences, Istanbul 34480, Turkey; erkut_ozturk@yahoo.com

**Keywords:** congenital heart disease, candidemia, intensive care unit

## Abstract

**Background:** Candida infections have become a significant cause of morbidity and mortality in pediatric cardiac intensive care units following congenital heart surgery, ranking among the most common causes of complications in this patient population. There is a paucity of information available regarding the epidemiology, clinical features, and risk factors associated with candidemia in this patient population. The present study evaluates the incidence of Candida bloodstream infections in pediatric cardiac intensive care units. **Methods:** The study was conducted retrospectively on cases of patients under the age of 18 who were admitted to the pediatric cardiac intensive care unit between 1 January 2021 and 1 January 2024. The isolated pathogens were recorded. A reanalysis was conducted on 36 patients with Candida bloodstream infections, with data pertaining to age, weight, cardiac pathologies, duration of mechanical ventilation, length of hospital stay, and antibiotic use being subjected to further examination. Each case was matched with two control patients based on age and date of surgery. The results were analyzed statistically. **Results:** A total of 36 cases of candidemia were identified and matched with 72 control cases. The incidence of candidemia was found to be 21.8 episodes per 1000 hospital admissions. The median age of patients with candidemia was four months. *Candida* species were identified in the blood cultures of 36 out of 1650 patients (0.21%). *Candida albicans* (*n* = 12, 33.3%), *Candida parapsilosis* (*n* = 16, 44.4%), *Candida glabrata* (*n* = 2, 5.5%), and other non-albicans *Candida* species (*n* = 6, 16.6%) were isolated. The mortality rate associated with Candida bloodstream infections was 61.1% (22/36). The following independent risk factors were identified as being associated with candidemia: a birth weight of less than 2500 g (OR: 3.2; 95% confidence interval (CI): 2.5–5; *p* = 0.009), a RACHS-1 score of 4 or above (OR: 2.1; 95% CI: 1.3–6; *p* = 0.01), cumulative antibiotic exposure of seven days or more (OR: 2.5; 95% CI: 2–10; *p* < 0.001), duration of central venous catheterization (CVC) of ≥14 days (OR: 6.1; 95% CI: 4–18; *p* < 0.001), mechanical ventilation dependency of ≥10 days (OR: 4.2; 95% CI: 3–11; *p* = 0.01), a requirement for total parenteral nutrition (OR: 9; 95% CI: 6–24; *p* < 0.001), and delayed sternal closure of ≥2 days (OR: 1.8; 95% CI: 1–4; *p* = 0.04). **Conclusions:** Postoperative candidemia represents a significant complication in pediatric patients with congenital heart disease (CHD), with different *Candida* species identified as a potential cause. The primary risk factors that contribute to the likelihood of a Candida bloodstream infection in these cases are a low birth weight, a high RACHS-1 score, dependence on mechanical ventilation, prolonged exposure to antibiotics, prolonged central venous catheter duration, delayed sternal closure, and total parenteral nutrition.

## 1. Introduction

Congenital heart diseases (CHDs) represent a significant public health concern in both developed and developing countries. The prevalence of these conditions is estimated to be 9 per 1000 live births, representing approximately one-third of all congenital defects [1,2]. Due to the wide range of anatomical and physiological variations in CHDs, surgical interventions are often required early in life to improve survival and quality of life. These procedures, although lifesaving, can also increase the risk of postoperative complications, including infections.

In addition, CHDs have a significant healthcare burden in terms of long-term medical expenses, the requirement for specialist multidisciplinary treatment, and morbidity and mortality.

CHDs are classified into various subclasses regarding the pathophysiology. The surgical approach may be corrective or palliative, depending on the specific problem. Furthermore, a number of underlying factors, including a low birth weight, genetic syndromes, and preoperative clinical conditions, can have a significant impact on the success of the surgery. Postoperative outcomes in these patients are influenced not only by surgical factors but also by perioperative management, infection control strategies, and the overall clinical status of the patient.

The frequency and severity of postoperative complications may be influenced by institutional factors, including infection monitoring systems, postoperative care standards, and the availability of pediatric cardiac specialists, in addition to patient-specific considerations.

Advancements in myocardial protection, surgical techniques, and innovations in intensive care unit monitoring systems have resulted in a reduction in morbidity and mortality rates. Furthermore, there have been alterations in the underlying causes of mortality and morbidity. Despite these improvements, post-surgical complications, particularly infections, remain a leading cause of adverse outcomes in pediatric cardiac surgery patients.

Bloodstream infections continue to represent a significant challenge in the context of pediatric cardiac intensive care units. Fungal bloodstream infections, predominantly caused by *Candida* species (spp.), represent a significant concern. Recent studies have highlighted an increasing trend in fungal infections in critically ill pediatric patients, emphasizing the need for early detection and targeted antifungal therapy [3,4,5,6,7].

Studies have reported that the prevalence of candidemia in pediatric intensive care units ranges between 10% and 15%, with even higher rates observed in cardiac intensive care units following congenital heart surgery [8,9,10]. The population of children with underlying CHD who have undergone cardiac surgery is characterized by heterogeneity and the challenges associated with its complexity. This population may be more susceptible to infection due to additional specific risk factors associated with certain medical or surgical comorbidities, including underlying diseases, immunodeficiency, delayed sternal closure, and the use of extracorporeal membrane oxygenation (ECMO) [8,9,10,11].

Furthermore, this susceptible group frequently needs several re-operations or staged surgical procedures, a fact which, over time, raises their total exposure to nosocomial risk factors. Prolonged hospital stays and frequent antibiotic treatment might disturb the natural flora and put patients at risk of opportunistic infections like candidemia.

A review of the literature reveals a paucity of studies examining the current incidence of candidemia and associated factors in pediatric patients with CHD who have undergone heart surgery. Moreover, the impact of fungal bloodstream infections on long-term clinical outcomes in this population remains underexplored, underscoring the need for further research. The objective of this study is to investigate the frequency of bloodstream infections associated with Candida species and the related risk factors in the pediatric cardiac intensive care unit of our heart center.

## 2. Methods

The study was conducted as a retrospective case-control study of patients under the age of 18 years who were admitted to the pediatric cardiac intensive care unit between 1 January 2021 and 1 January 2024. Our unit is a 39-bed level 3 cardiac center where all CHD, except for heart transplants, are monitored and treated.

Blood cultures were obtained from patients who required mechanical ventilation for more than 3 days and showed signs of infection according to the Centers for Disease Control and Prevention (CDC) criteria. Isolated pathogens were recorded. The isolation of *Candida* species from at least one blood culture was our definition of candidemia. Only the initial episode of candidemia was taken into consideration for the study if a patient experienced more than one incident during the study period. Control patients did not develop candidemia despite being admitted to the same PICU for CHD surgery at the same time (range up to ±30 days) and age (range ±10%) [12]. Particular attention was paid to critical clinical features such as single ventricle physiology and cyanosis. For every research patient, two matching controls were gathered.

Age, sex, the type of cardiac defect, and underlying comorbidities at admission, such as prematurity (for infants under one year old), major congenital malformations, genetic syndromes, primary immunodeficiency, chronic lung disease, renal failure, and the presence of liver or neurological disease, were among the pertinent baseline data gathered from medical records.

The data collection process was standardized using a structured case report form, and each variable was reviewed independently by two investigators to minimize data entry errors and enhance accuracy.

Cardiopulmonary bypass (CPB) time in minutes, the length of preoperative mechanical breathing, and the risk adjustment for congenital heart surgery (RACHS-1) score were among the pre-, peri-, and intraoperative factors [2].

A central venous catheter (CVC) for more than 48 h, thrombocytopenia (<100,000/mm^3^) at the time of PICU admission, the length of stay in the PICU, the duration of postoperative mechanical ventilation, the use of total parenteral nutrition (TPN) for more than 48 h, the development of chylothorax or the requirement for peritoneal dialysis, delayed sternal closure, or ECMO were all considered postoperative variables. If these indicators were detected within two weeks prior to the commencement of candidemia, just the existence of a CVC and the start of TPN were taken into consideration.

Additionally, we documented prior bloodstream infections (BSIs), which are defined as the isolation of bacteria from blood in patients exhibiting signs and symptoms of infection, as well as Candida colonization, which is defined as the isolation of *Candida* spp. from non-sterile locations in the absence of infection-related symptoms

To ensure consistency in diagnosis and reduce selection bias, inclusion criteria were strictly based on laboratory-confirmed candidemia episodes in conjunction with clinical signs of infection. The control group was matched carefully by date and age to minimize confounding variables.

Every detail pertaining to corticosteroid therapy, antimicrobial therapy, and antifungal prophylaxis was recorded, including the particular antimicrobial agent and usage duration. When calculating the overall cumulative antimicrobial therapy, several antibiotics administered on the same day were counted as multiple antibiotic days.

Catheter-related candidemia was defined according to the Infectious Disease Society of America guidelines [13], and crude mortality, defined as the rate of death within 30 days of candidemia, was assessed [14].

Culture replicates were performed by identifying proliferating yeast colonies using matrix-assisted laser desorption/ionization-time-of-flight [MALDI-TOF] Microflex LT/SH Smart MS (Bruker Daltonics, Bremen, Germany) and the MALDI-Biotyper Compass IVD 4. 2.90 database. All antifungal susceptibility testing of Candida strains isolated from blood samples was performed using Sensititre YeastOne (SYO) kits (Thermo Fisher Diagnostics, Landsmeer, The Netherlands), according to the manufacturer’s test instructions. After 24 h of incubation, MIC values were determined according to CLSI M27-A4 [15], M60-2nd ed. [16], and the European Committee on Antimicrobial Susceptibility Testing (EUCAST) v.10.0 [17].

This study was conducted in compliance with the Declaration of Helsinki and was approved by the University of Health Sciences Türkiye, Basaksehir Cam and Sakura City Hospital Local Ethics Committee (Approval Number: KAEK/2024.341).

### Statistical Analyses

Data were analyzed using SPSS Statistics 21. Categorical variables were compared using the chi-square or Fisher’s exact test depending on the expected frequencies, ensuring appropriate use of statistical assumptions for each test. Continuous variables were assessed for normality using the Shapiro–Wilk test, and, due to the non-normal distribution, comparisons were made using the Mann–Whitney U test. Descriptive statistics were reported as the median and interquartile range (IQR) for continuous variables, and as frequency and percentage for categorical variables. Variables that showed a *p* value < 0.1 in univariable analysis were selected for multivariable logistic regression to control for confounding factors and assess independent associations. Odds ratios (ORs) with 95% confidence intervals (CIs) were calculated for each independent predictor. A two-tailed *p* value < 0.05 was considered statistically significant.

## 3. Results

During the study period, 1650 cardiac operations were performed. During the study period, there were 21.8 occurrences of candidemia for every 1000 cardiac PICU patients. The study comprised 108 patients in total, 36 of whom had candidemia and 72 of whom were matched controls. Control patients did not develop candidemia despite being admitted to the same PICU for CHD surgery at the same time (range up to ±30 days) and age (range ±10%). Particular attention was given to critical clinical characteristics such as single-ventricle physiology, with patients selected to ensure similar proportions between the candidemia and control groups (38.8% vs. 36.1%, respectively). Efforts were also made to maintain comparable rates of cyanotic heart disease across both groups (58.3% vs. 50%, respectively). Among the most commonly observed defects in the study population were transposition of the great arteries (TGA), hypoplasia of the aortic arch, tetralogy of Fallot, ventricular septal defect, and hypoplastic left heart syndrome.


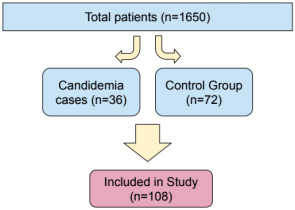


The median age of the patients with candidemia was 4 months (IQR 2–4) and the majority (55%) were male.

Candidemia remained for a median of 10 days (7–15 days) following treatment detection and developed for a median of 20 days [IQR 15–25] following surgery. The majority of cases (*n* = 28, 78%) had candidemia for at least 5 days. In 34 of 36 patients (94.4%), a CVC was already present at the commencement of candidemia, and 18 cases of candidemia (18/36, 50%) were recognized as catheter-related BSI. Underlying comorbidities were present in only a small percentage of individuals (*n* = 9, 25%). Six patients (16.7%) with candidemia showed signs of septic shock. A total of 13.8% of cases had concurrent endocarditis, 8.3% had concurrent peritonitis, and 8.3% had concurrent mediastinitis. The 30-day crude mortality rate for candidemia was 61.1% (22/36).

**Figure 1:**
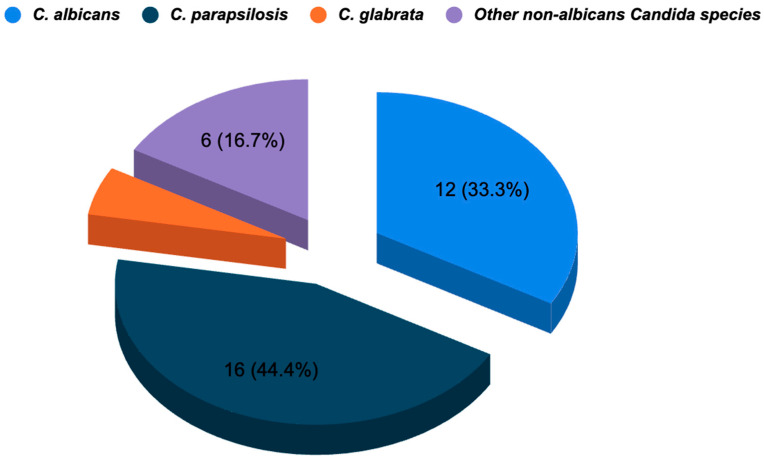
Distribution of *Candida* species in bloodstream infections.

Thirty-six out of 1650 patients (2.2%) had blood cultures that included *Candida* species. *Candida parapsilosis* (*n* = 16, 44.4%), *Candida albicans* (*n* = 12, 33.3%), *Candida glabrata* (*n* = 2, 5.6%), and additional non-albicans *Candida* species (*n* = 6, 16.7%) were isolated. (Figure 1).

All Candida strains were susceptible to amphotericin B. Susceptibility rates to echinocandins, voriconazole, and fluconazole were 97.2%, 85.9%, and 77.8%, respectively. *Candida albicans* isolates (*n* = 12) demonstrated susceptibility rates of 66.7% to both fluconazole and voriconazole, and 100% to echinocandins and amphotericin B. *Candida parapsilosis* isolates (*n* = 16) showed susceptibility rates of 81.3% to fluconazole, 93.8% to both voriconazole and echinocandins, and 100% to amphotericin B.

Additional findings revealed that patients who developed candidemia had longer hospital stays *(p* < 0.001) and higher postoperative support requirements, including ECMO (*p* < 0.001) and peritoneal dialysis (*p* = 0.03), further reflecting the severity of their clinical course.

First, univariate analysis was used to examine the factors linked to the emergence of candidemia in the cardiac PICU. A thorough comparison of the clinical and demographic features of the control and candidemia groups is shown in Table 1. In a number of variables, statistically significant differences were found. Notably, patients with candidemia had a significantly lower birth weight (*p* < 0.001), a higher incidence of RACHS-1 score ≥ 4 (*p* = 0.01), a longer central venous catheter duration (*p* < 0.001), and longer periods of postoperative mechanical ventilation (*p* < 0.001). The use and duration of TPN, presence of ECMO, chylothorax, peritoneal dialysis, delayed sternal closure, prior bacteremia, Candida colonization, and greater cumulative antibiotic exposure were also significantly associated with candidemia (all *p* < 0.05). Conversely, variables such as sex, presence of single ventricle physiology, cyanotic heart disease, preoperative ventilation days, CPB use and duration, thrombocytopenia, arrhythmias, acute kidney injury, LCOS, antifungal prophylaxis, and corticosteroid use did not differ significantly between the groups (*p* > 0.05). These results suggest that both patient-related and treatment-related factors contribute meaningfully to the risk of developing candidemia.

Subsequent multivariable logistic regression analysis identified the risk factors (Table 2) that remained independently associated with candidemia: a birth weight < 2500 g [OR: 3.2; 95% confidence interval (CI): 2.5–5; *p* = 0.009], RACHS-1 ≥ 4 [OR: 2.1; 95% CI: 1.3–6; *p* = 0.01], cumulative antibiotic exposure for ≥7 days [OR: 2.5; 95% CI: 2–10; *p* < 0.001], central venous catheter duration.

## 4. Discussion

In this study, the incidence of Candida bloodstream infections and the factors associated with candidemia in congenital heart surgery patients in a PICU over a three-year period were investigated. We found that candidemia is not uncommon in patients who have undergone congenital heart surgery. Control patients did not develop candidemia despite being admitted to the same PICU for CHD surgery at the same time (range up to ±30 days) and being of a similar age (range ±10%). The two groups were standardized based on clinical features that may play an important role in the development of candidemia, such as single-ventricle physiology and cyanotic heart disease. However, due to the high heterogeneity of surgical procedures and the limited number of patients undergoing each specific surgery type, subgroup standardization based on RACHS-1 surgical scores was not feasible. Similarly, standardization based on comorbidities might have allowed for a clearer interpretation of the results; however, the impact of these variables could not be fully anticipated at the study design stage. Nevertheless, the primary objective of this study was to identify clinical characteristics and risk factors associated with candidemia. Therefore, the variability observed between groups is considered a natural outcome of the study and is valuable for identifying potential risk factors. A low birth weight, a high RACHS-1 score, dependence on mechanical ventilation, prolonged exposure to antibiotics, prolonged duration of CVC, delayed sternal closure, and TPN were identified as independent risk factors for Candida bloodstream infection. Our study is one of the few studies reported in the literature focusing on this patient population.

Worldwide, invasive fungal diseases brought on by different fungal species are becoming a more significant issue. Specifically, the seventh most frequent cause of nosocomial sepsis in infants is now *Candida* species [16]. According to reports, the prevalence of fungal infections in children after congenital heart surgery ranges from 0.2% to 0.65%. A small number of patients are included in the research published in the literature, or they are case reports [18,19,20,21].

Motta and colleagues found a frequency of 0.7 cases per 1000 patient-days [22], while Kahan et al. reported an incidence of 6.3 cases per 1000 PICU hospitalizations after heart surgery [12] in their research including PICU patients. The rate in our study was 21.8 cases for every 1000 PICU admissions. The complexity and heterogeneity of patients may have an impact on the increased rate.

*Candida albicans, Candida parapsilosis, Candida tropicalis, Candida glabrata,* and other non-albicans *Candida* species can all cause candidemia. According to a recent study, out of 1056 patients monitored in the PICU, 137 (12.9%) had blood cultures showing *Candida* species. *Candida albicans* (*n* = 50, 36.5%), *Candida parapsilosis* (*n* = 20, 14.6%), *Candida tropicalis* (*n* = 8, 5.8%), *Candida glabrata* (*n* = 5, 3.7%), and additional non-albicans *Candida* species (*n* = 54, 39.4%) were recognized as these species [5]. According to a different study, almost half of the cases of candidemia were caused by species of *Candida* other than *Candida albicans*. The most commonly isolated species was found to be *C. parapsilosis* [12]. The most often isolated species in our investigation was *C. parapsilosis*, which is in line with CVC’s function as a possible entry gateway. Similar distributions of *Candida* species have been found in other investigations, indicating a global shift toward non-albicans *Candida* species that cause candidemia.

A rise in antifungal resistance, especially in non-albicans species, has also been documented in recent reviews. From 4.1% in the USA to 32%, 33%, and 63% in India, Italy, and South Africa, respectively, the prevalence of azole-resistant *C. parapsilosis* varies significantly by geographic location [3,12,23,24,25,26,27,28]. The susceptibilities in our study were 77.7% for fluconazole, 86% for voriconazole, and 97.2% for amphotericin B.

Cardiac surgery differs significantly from other types of surgery because of the frequent use of cardiopulmonary bypass and hypothermia, the complexity of treatments, the long duration of surgery, the prolonged stay in the PICU, and the use of many invasive therapeutic procedures. Cardiac surgical intensive care units differ from conventional pediatric ICUs in that they involve more invasive interventions, longer lengths of stay, and a more complex patient population. These factors may contribute to the increased frequency of risk factors such as prolonged central venous catheter (CVC) duration, mechanical ventilation, and total parenteral nutrition (TPN) use. In this context, delayed sternal closure, along with these variables, may represent characteristics specific to the cardiac ICU setting. Numerous research on the independent risk factors linked to candidemia in adult and pediatric populations have been carried out, and these risk factors may raise the prevalence of fungal infections in this patient group [29,30,31,32,33,34]. Male sex (OR: 6.2; 95% CI: 1.9–20.3; *p* = 0.002), the need for total parenteral nutrition or peritoneal dialysis (OR: 6.1; 95% CI: 2–18.8; *p* = 0.001), cumulative antibiotic exposure for ≥4 days (OR: 4.3; 95% CI: 1.3–14.6; *p* = 0.02), and delayed sternal closure ≥2 days (OR: 3.2; 95% CI: 1–11.2; *p* = 0.05) were all independent risk factors linked to candidemia in the Kahan studies [12].

In the study by Motta and colleagues, thrombocytopenia, the use of acid suppression treatment, and a risk adjustment for congenital heart surgery (RACHS-1) category of ≥3 were risk variables that were independently linked to candidemia [20]. A low birth weight, a high RACHS-1 score, reliance on mechanical ventilation, extended antibiotic exposure, prolonged central venous catheterization, delayed sternal closure, and use of total parenteral nutrition were among the factors that our study examined and found to be independent risk factors.

Moreover, our findings suggest that modifiable risk factors such as the duration of central venous catheterization and cumulative antibiotic exposure could be targeted through stricter antimicrobial stewardship programs and catheter management protocols.

Implementing bundled care approaches and developing unit-specific infection control policies may further enhance outcomes by standardizing preventive strategies against nosocomial fungal infections.

Candidemia is known to be associated with increased mortality and morbidity. In the study by Karaağaç and colleagues, the mortality rate for Candida bloodstream infections was 11.6% (16/137) [9]. There are series where this rate has increased up to 50% [12,20]. The mortality rate for candidemia in our study was 61.1%.

### Limitations

The main limitation of this study is that it was carried out on a limited number of cases, in a single center, and in a retrospective manner. The second limitation is that the diagnosis of candidemia was made solely on the basis of a positive blood culture, and the number of patients diagnosed with CVC-associated infection was determined by matching central and peripheral cultures.

## 5. Conclusions

Postoperative candidemia is a serious complication in children with congenital heart disease (CHD) and is associated with significant morbidity and mortality. Our study highlights the high prevalence of Candida bloodstream infections in the pediatric cardiac intensive care unit, emphasizing the need for early identification of risk factors and targeted preventive measures. The identification of *C. parapsilosis* as the most frequently isolated species suggests the critical role of central venous catheters as a primary source of infection, reinforcing the necessity for stringent catheter management protocols.

The major risk factors contributing to Candida bloodstream infection include a low birth weight, a high RACHS-1 score, prolonged mechanical ventilation, extended antibiotic exposure, prolonged central venous catheter duration, delayed sternal closure, and total parenteral nutrition. These findings underscore the importance of a multidisciplinary approach in managing postoperative pediatric cardiac patients, integrating infectious disease specialists, intensivists, and cardiothoracic surgeons to mitigate infection risks. Furthermore, the increasing trend of antifungal resistance, particularly among non-albicans *Candida* species, necessitates continuous surveillance of antifungal susceptibility patterns to guide appropriate treatment strategies.

Future prospective, multicenter studies are needed to validate our findings and explore additional modifiable factors that may improve outcomes.

## Figures and Tables

**Table 1:** Demographic and clinical variables for patients with candidemia and control patients.

Variables	Candidemia (*n* = 36)	Control (*n* = 72)	*p*
Comorbidities	9 (25)	4 (5.5)	0.015
Age, months	4 (2–6)	6 (3–9)	0.680
Weight, kg	5.1 (3–8)	7.2 (6–8.4)	0.040
Birth weight, kg			<0.001
≥2.5	24 (67)	68 (94.5)	
<2.5	12 (33)	4 (5.5)	
Male	20 (55)	38 (53)	0.990
Single ventricle physiology	14 (38.8)	26 (36.1)	0.990
Cyanotic heart disease	21 (58.3)	36 (50)	0.860
Duration of preoperative mechanical ventilation, days	2 (0–4)	1 (0–2)	0.990
CPB use	33 (91.6)	68 (94.5)	0.900
CPB time, min	80 (65–95)	70 (60–80)	0.450
RACHS-1 ≥ 4	18 (50)	16 (22.2)	0.01
Central venous catheter duration, days	21 (15–28)	7 (5–10)	<0.001
Thrombocytopenia (<100,000/mm^3^) at ICU admission	20 (55.5)	35 (48.6)	0.780
Duration of postoperative mechanical ventilation, days	14 (10–18)	3 (1–5)	<0.001
TPN	22 (61.1)	11 (15.2)	<0.001
Duration of TPN, days	10 (6–14)	2 (1–3)	<0.001
ECMO	4 (11.1)	-	<0.001
Arrhythmias	3 (8.3)	7 (9.7)	0.810
Acute kidney injury	6 (16.6)	8 (11.1)	0.540
LCOS	11 (30.5)	18 (25)	0.990
Chylothorax	7 (19.4)	1 (1.3)	0.001
Peritoneal dialysis	6 (16.6)	4 (5.5)	0.03
Delayed sternal closure ≥2 days	15 (41.6)	5 (6.9)	<0.001
Prior bacteremia	9 (25)	6 (8.3)	0.04
*Candida* colonization	9 (25)	1 (1.3)	0.008
Any antimicrobial agent exposure	33 (91.6)	36 (50)	<0.001
Antimicrobial agents used ≥3	14 (38.8)	5 (6.9)	<0.001
Cumulative antibiotic treatment duration	10 (7–14)	5 (3–7)	<0.001
Antifungal prophylaxis	2 (5.5)	1 (1.3)	0.130
Use of corticosteroids	7 (19.4)	11 (15.2)	0.870
ICU stay(days)	28 (21–35)	5 (3–7)	<0.001
Post-op hospital stay (days)	40 (32–50)	12 (8–14)	<0.001
Mortality	22 (61.1)	5 (6.9)	<0.001

Median (IQR) *n* (%). CPB: cardiopulmonary bypass, RACHS-1: risk adjustment for congenital heart surgery, ICU: intensive care unit, TPN: total parenteral nutrition, ECMO: extracorporeal membrane oxygenation, LCOS: low cardiac output syndrome.

**Table 2:** Multivariate logistic regression analysis of factors associated with the development of candidemia.

Variables	*p*	Odds Ratio	95% Confidence Interval
Birth weight < 2500 g	0.02	3.2	2.5–5
RACHS-1 ≥ 4	0.01	2.1	1.3–6
Central venous catheter duration ≥14 days	<0.001	6.1	4–18
Mechanical ventilation dependency ≥10 days	0.01	4.2	3–12
Total parenteral nutrition	<0.001	9	6–24
Cumulative antibiotic exposure for ≥7 days	<0.001	2.5	2–9
Delayed sternal closure ≥2 days	0.04	1.8	1–4.5

RACHS-: risk adjustment for congenital heart surgery.

## Data Availability

All data used in this study can be obtained from the corresponding author upon request.
